# Associations of Prenatal Nicotine Exposure and the Dopamine Related Genes *ANKK1* and *DRD2* to Verbal Language

**DOI:** 10.1371/journal.pone.0063762

**Published:** 2013-05-15

**Authors:** John D. Eicher, Natalie R. Powers, Kelly Cho, Laura L. Miller, Kathryn L. Mueller, Susan M. Ring, J. Bruce Tomblin, Jeffrey R. Gruen

**Affiliations:** 1 Department of Genetics, Yale University School of Medicine, New Haven, Connecticut, United States of America; 2 Departments of Epidemiology and Public Health, Yale University School of Medicine, New Haven, Connecticut, United States of America; 3 Division of Aging, Brigham and Women’s Hospital, Harvard Medical School, Boston, Massachusetts, United States of America; 4 Massachusetts Veterans Epidemiology Research and Information Center, Boston, Massachusetts, United States of America; 5 School of Social and Community Medicine, University of Bristol, Bristol, United Kingdom; 6 Departments of Speech, Pathology, and Audiology, University of Iowa, Iowa City, Iowa, United States of America; 7 Departments of Pediatrics and Investigative Medicine, Yale Child Health Research Center, Yale University School of Medicine, New Haven, Connecticut, United States of America; East Carolina University, United States of America

## Abstract

Language impairment (LI) and reading disability (RD) are common pediatric neurobehavioral disorders that frequently co-occur, suggesting they share etiological determinants. Recently, our group identified prenatal nicotine exposure as a factor for RD and poor reading performance. Using smoking questionnaire and language data from the Avon Longitudinal Study of Parents and Children, we first determined if this risk could be expanded to other communication disorders by evaluating whether prenatal nicotine exposure increases risk for LI and poor performance on language tasks. Prenatal nicotine exposure increased LI risk (OR = 1.60; p = 0.0305) in a dose-response fashion with low (OR = 1.25; p = 0.1202) and high (OR = 3.84; p = 0.0002) exposures. Next, hypothesizing that the effects of prenatal nicotine may also implicate genes that function in nicotine related pathways, we determined whether known nicotine dependence (ND) genes associate with performance on language tasks. We assessed the association of 33 variants previously implicated in ND with LI and language abilities, finding association between *ANKK1*/*DRD2* and performance on language tasks (p≤0.0003). The associations of markers within *ANKK1* were replicated in a separate LI case-control cohort (p<0.05). Our results show that smoking during pregnancy increases the risk for LI and poor performance on language tasks and that *ANKK1*/*DRD2* contributes to language performance. More precisely, these findings suggest that prenatal environmental factors influence *in utero* development of neural circuits vital to language. Our association of *ANKK1*/*DRD2* further implicates the role of nicotine-related pathways and dopamine signaling in language processing, particularly in comprehension and phonological memory.

## Introduction

Language impairment (LI) and reading disability (RD) have prevalences of 5–8% and 5–17%, respectively, in schoolchildren [1]–[2], and together comprise the majority of learning disabilities. LI and RD are characterized by difficulty in the understanding and/or utilization of verbal and written language, respectively, despite normal development and adequate educational opportunity [1]–[2]. LI and RD are related disorders, as both involve deficits in the integration and utilization of communicative tools. Impaired phonological skills have been implicated in both LI and RD [1]–[7]. LI and RD are frequently comorbid; as children with LI are at higher risk of developing RD than their typically developing peers [1]–[2], [8]. The degree of relatedness and the frequent comorbidity of LI and RD indicate they may share risk factors. Twin and family studies have shown that both LI and RD have a significant genetic component, with heritability estimates of 45–73% and 54–84%, respectively [8]–[10]. However, specific environmental and genetic risk factors for LI and RD, and the extent to which they are shared between the two disorders, remain largely unknown.

One possible environmental risk factor for LI and RD is exposure of the developing fetus to toxins and substances *in utero* via the maternal environment and behavior, specifically smoking or nicotine exposure. The harm of prenatal nicotine exposure has been well-documented [11]–[13]. Despite this, studies estimate 14–37% of women smoke during pregnancy [14]. Prenatal nicotine exposure is a risk factor for several neurobehavioral conditions such as Attention Deficit-Hyperactivity Disorder (ADHD), learning disabilities, and substance abuse [15]–[17]. Some studies have expanded these findings to RD, LI, and neurocognition [18]–[19], [21]; while others have argued that nicotine variables may capture factors not adequately controlled for in statistical models, such as socioeconomic status [20]–[21]. Our recent work showed an association between prenatal nicotine exposure and poor reading performance in schoolchildren, after adjustment for a wide range of confounders, including socioeconomic status, type of school attended, birth weight, and gestational age [22]. However, further study is necessary to determine whether prenatal nicotine exposure also influences language abilities and LI.

The implication of prenatal nicotine exposure in communication performance raises the question of how this exposure exerts its effects. One possibility is that genetic variants previously associated with nicotine dependence (ND) and nicotine-related pathways may have pleiotropic effects. That is, genetic variants that predispose individuals to ND may also contribute to communication processes. Studies of ND have revealed that it has a significant genetic component and identified several candidate genes, including *DRD2*, *ANKK1*, *CHRNA4*, and *CHRNB2*. Many of these genes are involved in neuronal signaling pathways, including the cholinergic and dopaminergic neurotransmitter pathways. The implication of various signaling pathways further suggests that variation in these genes may affect multiple cerebral processes, such as addiction, language, and reading. Several of these ND genes, including *BDNF*, *DRD2*, and *ANKK1*, have been associated with neurobehavioral phenotypes [23]–[24]. *ANKK1* and *DRD2* have been associated with autism, executive functioning, and verbal ability [25]–[26]. However, these reports are few in number, and replication in larger cohorts is needed.

The present study expands on our previous work to examine prenatal nicotine exposure and its related pathways with regard to LI and its associated language domains. First, we analyze the relationship of prenatal nicotine exposure with performance on language tasks and LI. Due to nicotine’s detrimental effects on brain function, we hypothesize that prenatal smoking exposure will also be a risk factor for poor language performance and LI. Second, we assess whether known ND variants contribute to language abilities. ND genes have known neurological functions, particularly in neuronal signaling; therefore, we also hypothesize ND variants associate with language performance and LI.

## Materials and Methods

### Subjects

The Avon Longitudinal Study of Parents and Children (ALSPAC) is a population-based, birth cohort in Avon, United Kingdom. Subjects were recruited before birth, resulting in a total of 15,458 fetuses, of whom 14,701 were alive at 1 year of age. Recruitment, participants, and study methodologies are described in detail elsewhere (http://www.bristol.ac.uk/alspac) [27]–[28]. 7170 subjects completed language measures at age 8 years. Subjects with IQ ≤75 on the Wechsler Intelligence Scale for Children (WISC-III) Total IQ were excluded from the present study [29]. To prevent population stratification in genetic analyses, subjects of non-European descent were also removed. Additionally, samples with genotyping call rate <0.80 were excluding from analyses, leaving a final sample size of 5579 individuals. Ethical approval was obtained from ALSPAC Ethics and Law Committee, Local UK Research Ethics Committees, and Yale Human Investigation Committee.

### ALSPAC Language Measures

Language measures were collected during clinical interviews at age 8 years. An adaptation of the Nonword Repetition Task (NWR), in which subjects repeated recordings of nonwords, was used to assess short-term phonological memory and processing abilities [30]. Children also completed the Wechsler Objective Language Dimensions (WOLD) verbal comprehension task at age 8 years [31], where they answered questions about a paragraph read aloud by an examiner describing a presented picture. We focused on these measures because individuals with LI are known to consistently perform poorly on NWR and WOLD comprehension tasks, and these tasks are commonly used in genetic and epidemiologic studies of LI [32]–[33]. Z-scores were calculated for each subject on each individual measure, and to capture deficits in two of the primary domains of LI, the average z-score of NWR and WOLD comprehension tasks was calculated. To assess the risk imparted to severe LI, we defined LI cases as scoring ≥2.0 standard deviations below sample means on either task.

### Exposure and Covariate Variables

Questionnaires for smoking frequency and cigarette brand were completed by mothers at gestational age 8, 18, and 32 weeks and at 8 weeks following birth. Although cigarettes contain thousands of compounds, nicotine is the most prevalent, pharmacologically active ingredient that is likely responsible for smoking’s deleterious effects. Therefore, we calculated the level of nicotine exposure for each time point based upon the nicotine content of the cigarette brands smoked. Because of limited power to divide nicotine exposure into trimesters, we used the maximum nicotine exposure to derive prenatal nicotine exposure [22], [34]–[35]. First, prenatal nicotine exposure was dichotomized into exposed and non-exposed groups. To examine dose-response, prenatal nicotine exposure was further categorized into three groups: no exposure (0 mg*day^−1^), low exposure (≤17 mg*day^−1^), and high exposure (>17 mg*day^−1^) [36]. 17 mg was chosen as it is the average amount of nicotine in one pack of cigarettes.

Due to the interdependence between overall cognition and communication, subjects with WISC-III Total IQ scores ≤75 were excluded from analysis [37]. To further control for the effects of IQ, WISC-III Performance IQ scores were included as a covariate in analyses [29]. Performance IQ was chosen to prevent controlling for language abilities captured by Verbal and Total IQ scores. In addition to Performance IQ, we adjusted for the following 11 covariates to control for known confounding relationships with language: mother’s age at delivery, maternal prenatal alcohol consumption [38], maternal social class, child-parent interaction time, mother’s attendance at antenatal classes, sex, ADHD status, school type, gestational age, birthweight, and resuscitation status [39] (Table S1).

### Statistical and Genetic Analyses

First, SAS 9.2 was used to statistically analyze the association of prenatal nicotine exposure with language performance in the ALSPAC cohort. Dichotomized prenatal nicotine exposure status was examined first, followed by dosage categories. For quantitative measures, we fitted crude linear regression models, with prenatal nicotine exposure as the predictor for each language outcome. Next, multivariable regression models adjusted for covariates were used to identify specific effects of prenatal nicotine exposure. We used logistic regression models to fit prenatal nicotine exposure and covariates for each dichotomized language measure. Odds ratios (OR) were calculated for exposed/non-exposed, then for the low and high dosage categories.

Next, 33 single nucleotide polymorphisms (SNPs) in 12 genes, previously implicated in ND, nicotine pathways, and/or substance dependencies, were genotyped on the Sequenom platform (San Diego, CA), following the manufacturers guidelines at the Yale Center for Genome Analysis (Orange, CT) (Table 1). 32 of the 33 ND variants had call rates ≥90%, were biallelic, had minor allele frequencies ≥0.01, and were in Hardy-Weinberg equilibrium (p≥0.001). To correct for the 32 genetic association tests performed in the ALSPAC cohort, Bonferroni correction was applied to adjust for multiple testing (α = 0.05/32 = 1.56×10^−3^). Since these ND variants have a prior relationship with nicotine and/or addiction, we created a subsample of subjects not exposed to nicotine and repeated associations to avoid possible confounding.

**Table 1:** Nicotine dependence (ND) markers genotyped in the ALSPAC sample.

Variant	Gene	Location	MAF	Variant	Gene	Location	MAF
rs2072660	*CHRNB2*	1q21.3	0.240	rs10893365	*PKNOX2*	11q24.2	0.171
rs2072661	*CHRNB2*	1q21.3	0.244	rs10893366	*PKNOX2*	11q24.2	0.168
rs12466358	*CHRND*	2q31	0.253	rs11220015	*PKNOX2*	11q24.2	0.174
rs13277254	*CHRNB3*	8p21	0.212	rs11602925	*PKNOX2*	11q24.2	0.176
rs4950	*CHRNB3*	8p21	0.214	rs12284594	*PKNOX2*	11q24.2	0.170
rs6474413	*CHRNB3*	8p21	0.214	rs1426153	*PKNOX2*	11q24.2	0.174
rs4075274	*NTRK2*	9q21.33	0.434	rs750338	*PKNOX2*	11q24.2	0.227
rs2030324	*BDNF*	11p14.1	0.469	rs1051730	*CHRNA3*	15q25	0.329
rs4274224	*DRD2*	11q23.1	0.493	rs1317266	*CHRNA3*	15q25	0.226
rs4648318	*DRD2*	11q23.1	0.239	rs578776	*CHRNA3*	15q25	0.281
rs7131056	*DRD2*	11q23.1	0.425	rs6495308	*CHRNA3*	15q25	0.231
rs6278	*DRD2*	11q23.1	0.153	rs8034191	*LOC123688*	15q25	0.331
rs11604671	*ANKK1*	11q23.1	0.488	rs16969968	*CHRNA5*	15q25	REMOVED
rs1800497	*ANKK1*	11q23.1	0.197	rs2229959	*CHRNA4*	20q13.33	0.113
rs2734849	*ANKK1*	11q23.1	0.485	rs2236196	*CHRNA4*	20q13.33	0.252
rs4938013	*ANKK1*	11q23.1	0.321	rs2273504	*CHRNA4*	20q13.33	0.162
rs7118900	*ANKK1*	11q23.1	0.185				

Abbreviations: ND, nicotine dependence; MAF, minor allele frequency.

Associated variants were then examined in the Iowa LI cohort. The Iowa LI cohort is comprised of 219 LI cases and 209 sex- and age-matched, unrelated controls collected at the University of Iowa. Subjects completed various language measures, including the Peabody Picture Vocabulary Test (PPVT) and NWR, which were used to derive a composite language score, which was dichotomized into case-control status at −1.14 standard deviations [40]. Single marker analysis in both cohorts was performed with linear and logistic regression under additive models using SNP & Variation Suite (SVS) v7.6.4 (Golden Helix, Bozeman, MT). Haplotype regions were constructed following the 4-gamete rule using HaploView v4.2, and haplotype association tests were performed using PLINK v1.07.

## Results

### Prenatal Nicotine Exposure and Language

In the ALSPAC sample, subjects exposed to prenatal nicotine performed on average 4.75–5.39% worse on language measures compared to non-exposed subjects (Table 2). When separated into nicotine dosage categories, those exposed to high levels of prenatal nicotine performed on average the worst on all measures compared to low (ranging from 6.20–7.95% worse) and no exposure (ranging from 9.63–11.58%) groups (Table 2).

**Table 2:** Descriptive statistics of language scores among exposure groups.

	Non-smoking		Any Exposure		Low		High	
	N	Mean(SD)	N	Mean(SD)	N	Mean(SD)	N	Mean(SD)
NWR	4720	7.37(2.43)	758	7.02(2.48)	615	7.10(2.45)	143	6.66(2.56)
Comprehension	4724	7.60(1.91)	760	7.19(1.93)	617	7.30(1.93)	143	6.72(1.88)

Abbreviations: SD, standard deviation; NWR, nonword repetition.

Crude linear regression analyses comparing groups exposed to prenatal nicotine to the non-exposed groups showed that prenatal nicotine exposure is associated with performance on NWR and comprehension tasks (p≤0.0002) (Table 3). After adjusting for covariates, the association with average performance on the NWR/comprehension tasks persisted (p = 0.0262), while there was a trend with the NWR task (p = 0.0799). Crude analyses for exposure dosage showed a deleterious effect of prenatal nicotine exposure on NWR and comprehension tasks (p≤0.0002) (Table 4). After covariate adjustment, there was a negative effect of high dose of prenatal nicotine exposure on comprehension (p = 0.0011) and average performance on NWR/comprehension (p = 0.0011), with trend toward a negative effect of high exposure for the NWR task alone (p = 0.0729).

**Table 3:** Effects of any prenatal nicotine exposure on language performance.

	Crude Model			Adjusted Model		
	Exposed		Overall	Exposed		Overall
Measure	Beta	p-value	p-value	Beta	p-value	p-value
NWR	−0.14	0.0002	0.0002	−0.09	0.0799	0.0799
Comprehension	−0.21	<0.0001	<0.0001	−0.08	0.1123	0.1123
Avg NWR Comp	−0.18	<0.0001	<0.0001	−0.09	0.0262	0.0262

Abbreviations: NWR, nonword repetition; Avg NWR Comp. average of z-scores of nonword repetition and verbal comprehension tasks.

**Table 4:** Effects of prenatal nicotine dosage on language performance.

	Crude Model					Adjusted Model				
	Low		High		Overall	Low		High		Overall
Measure	Beta	p-value	Beta	p-value	p-value	Beta	p-value	Beta	p-value	p-value
NWR	−0.11	0.0102	−0.29	0.0006	0.0002	−0.07	0.2174	−0.22	0.0729	0.1085
Comprehension	−0.16	<.0002	−0.46	<0.0001	<0.0001	−0.02	0.7426	−0.47	0.0002	0.0011
Avg NWR Comp	−0.13	<0.0001	−0.37	<0.0001	<0.0001	−0.05	0.2868	−0.35	0.0003	0.0011

Abbreviations: NWR, nonword repetition; Avg NWR Comp. average of z-scores of nonword repetition and verbal comprehension tasks.

In ALSPAC, LI had a prevalence of 4.90%, which is consistent with estimates in the general population [1]–[2]. Exposure to prenatal nicotine increased risk for LI, after controlling for covariates (OR = 1.60 [1.04–2.45]; p = 0.0305) (Table 5). Risk of developing LI occurred in a dose response fashion with low (OR = 1.25 [0.76–2.04]; p = 0.1202) and high (OR = 3.85 [1.87–7.94]; p = 0.0009) prenatal nicotine exposure levels (Table 6).

**Table 5:** LI risk based on prenatal nicotine exposure.

Crude Model		Adjusted Model	
OR	p-value	OR	p-value
1.77 (1.31–2.40)	0.0002	1.60 (1.04–2.45)	0.0305

Abbreviation: OR, odds ratio.

**Table 6:** LI risk based on prenatal nicotine dosage.

Crude Model				Adjusted Model			
Low OR	p-value	High OR	p-value	Low OR	p-value	High OR	p-value
1.43 (1.00–2.05)	0.2514	3.33 (2.02–5.52)	<0.0001	1.25 (0.76–2.04)	0.1202	3.85 (1.87–7.94)	0.0009

Abbreviation: OR, odds ratio.

### Association of ND Markers to Language

**Figure 1:**
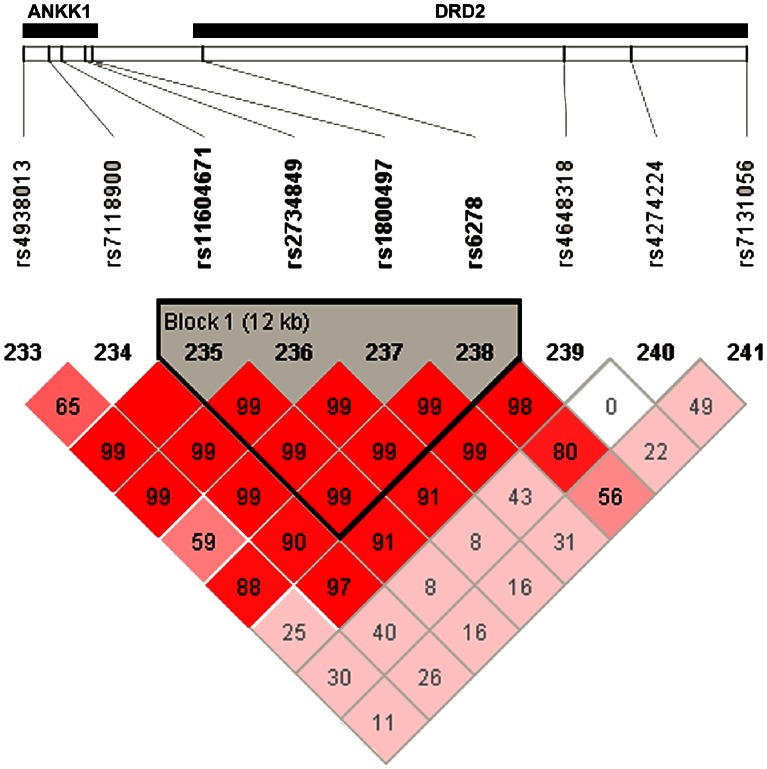
Linkage disequilibrium between *ANKK1* and *DRD2*. Linkage disequilibrium (LD), as measured by D’, among markers in the *ANKK1* and *DRD2* genes. There is a 12kb haplotype block spanning the two genes (markers: rs11604671, rs2734849, rs1800497, and rs6278).

Single-marker analysis revealed associations between SNPs within *ANKK1* and language performance as measured by the average z-score on NWR/comprehension tasks (p≤1.9×10^−3^) (Table 7). Haplotype associations were similar, showing association between a haplotype containing *ANKK1* and *DRD2* markers and language performance (Table 8). This haplotype included a majority of the significant SNPs from single marker analysis, suggesting these markers captured the same variability in the locus. Interestingly, the *ANKK1* haplotype block contained a marker in the *DRD2* gene located adjacent to *ANKK1* (rs6278) (Figure 1). These associations persisted when examined in ALSPAC subjects not exposed to prenatal nicotine (Table 7). There was no evidence of interaction between prenatal nicotine exposure and ND variants in the ALSPAC sample. Associations of SNPs within *ANKK1* (rs2734849 and rs11604671) were replicated in the Iowa LI cohort with LI case-control status (OR = 1.4 [1.1–2.0]; p≤7.41×10^−3^) (Table 9).

**Table 7:** Single marker genetic associations with average of Nonword Repetition and Verbal Comprehension tasks.

Variant	Gene	p-value	No SMK p-value^a^
rs2734849	*ANKK1*	2.0×10^−4^	1.9×10^−4^
rs11604671	*ANKK1*	2.3×10^−4^	2.2×10^−4^
rs4938013	*ANKK1*	1.2×10^−3^	6.8×10^−4^
rs7118900	*ANKK1*	1.2×10^−3^	1.2×10^−3^
rs1800497	*ANKK1*	1.9×10^−3^	2.6×10^−3^
rs6278	*DRD2*	8.8×10^−3^	1.7×10^−2^

a No SMKg p-value refer to associations in cohort of subjects not exposed to prenatal nicotine.

**Table 8:** Haplotype Association of *ANKK1*/*DRD2* with average of Nonword Repetition and Verbal Comprehension tasks.

Variants	Genes	Haplotype	Beta	p-value
rs11604671, rs2734849, rs1800497, rs6278	*ANKK1*/*DRD2*	ACCG	0.053	3.2×10^−4^

**Table 9:** Replication of genetic associations in Iowa LI cohort.

Variant	Gene	Trait	p-value	OR
rs11604671	*ANKK1*	Case-Control	3.84×10^−3^	1.4 (1.1–2.0)
rs2734849	*ANKK1*	Case-Control	7.41×10^−3^	1.4 (1.1–2.0)
rs1800497	*ANKK1*	Case-Control	1.9×10^−2^	1.5 (1.1–2.1)
rs11604671	*ANKK1*	GORT Comp	3.3×10^−2^	N/A
rs11604671	*ANKK1*	PPVT	2.5×10^−2^	N/A

Abbreviations: OR, odds ratio; GORT Comp, Gray Oral Reading Test Comprehension; PPVT, Peabody Picture Vocabulary Test.

## Discussion

Our investigation examined the effects of prenatal nicotine exposure and nicotine-related genetic variants on LI and performance on language tasks. We found increased risk of LI and poor performance on language tasks in subjects exposed to prenatal nicotine. In addition, there was a genetic association between single markers within *ANKK1* and a haplotype spanning *ANKK1*/*DRD2* and language performance, further implicating nicotine-related and dopamine pathways in language. These findings show the importance of the prenatal environment and dopamine to language and cognitive development.

### Prenatal Nicotine Exposure and Language

We found an association of prenatal nicotine exposure on language performance and LI, after adjusting for known covariates, such as socioeconomic status, type of school attended, and parent interaction. This relationship appears to be specific to language skills and independent of overall cognitive skills, as Performance IQ was accounted for in all final models. These results expand upon our previous findings, showing the detrimental effects of prenatal nicotine exposure on phonology, reading fluency, reading comprehension, and reading accuracy. These components are foundational to the development of reading and language skills in children. Our previous study found that deficits in reading comprehension similar to the ones we found in verbal comprehension, suggesting prenatal nicotine exposure exerts an effect on how children ascertain meaning in verbal and written language.

The negative effects of prenatal nicotine exposure on reading and language may reflect changes in gene expression resulting from epigenetic modifications due to the nicotine exposure [41]. Future studies should examine how nicotine exposure interacts with genes associated with communication, such as *DCDC2*, *KIAA0319*, and *FOXP2*, and their epigenetic regulation. One investigation demonstrated the contribution of 5′ regions marked by acetylated H3 histones in *KIAA0319* to RD, suggesting the importance of epigenetic regulation to language [42]. Epigenetic studies in combination with neurotoxicological studies should be explored to determine whether and how nicotine exposure alters gene expression and cellular function.

In addition to possible changes directly to gene regulation, mouse and rat models have shown that prenatal nicotine exposure permanently affects neurochemical signaling pathways, including dopaminergic pathways, and alter their developmental trajectories over the lifespan [43]–[45]. Animals exposed to gestational nicotine have higher dopamine turnover in the frontal cortex [44], [46]. These results from animal models, in conjunction with our findings implicating prenatal nicotine and dopamine signaling in LI, suggest that deficits and permanent changes in dopamine activity resulting from exposure and genetic variants have a substantial influence upon language skills and development. These implications, however, still must be explored and confirmed in human studies, possibly through magnetic resonance spectroscopy (MRS). MRS would permit *in vivo* monitoring of dopamine signaling in the human brain. MRS could specifically interrogate the influence of prenatal nicotine exposure on dopaminergic signaling, gene expression, and language in the same subjects.

### *ANKK1*/*DRD2* and Language

Single marker and haplotype analyses showed association between language performance and *ANKK1*-*DRD2*. In addition to past associations with ND, *ANKK1* and *DRD2* have been associated with other neurobehavioral traits including alcohol dependence, reinforcement learning, working memory, and executive function [47]–[51]. Dopamine is a key neurotransmitter in the corticostriatal system that subserves procedural and reinforcement learning. Animal and human studies, using dopamine agonists and/or antagonists, show that alterations in dopamine receptor function change reinforcement learning [52]. Recently, reinforcement learning was shown to be associated with individual differences in language in a task influenced by dopamine signaling [53]–[54]. Additionally, past studies have associated *ANKK1*/*DRD2* to working memory. Working memory is directly associated with language skills in children, and in fact, impairments in working memory have been proposed to play a direct role in the development of language deficits seen in children with LI [55]–[56]. Changes in dopaminergic function, whether from genetic predisposition (*ANKK1*/*DRD2*) or environmental exposure (prenatal nicotine), yield alterations in working memory and reinforcement learning. These changes, which arise via permanent alterations in dopamine function, appear to then influence language development as well as other neurobehavioral domains, including nicotine and substance use.

Despite the wide range of literature examining *ANKK1*/*DRD2* and neurobehavioral traits, there have been limited reports examining the role of *ANKK1* and *DRD2* specifically in language and language-related domains. Beaver et al. reported an association between *DRD2* and performance on an abbreviated form of the PPVT [26]. The PPVT is a standardized measure of expressive and receptive vocabulary, which may be analogous to deficits measured in our verbal and reading comprehension tasks, although the tasks in this study measure higher order cognitive processing. Our findings expand the role of *ANKK1* and *DRD2* from known effects on working memory, reinforcement learning, and predisposition to nicotine use to now include verbal language. Additionally, these findings point to a role for dopamine as a mechanism in processes involved in language development. In this regard, these findings and the implications of prenatal nicotine exposure on brain neurochemistry support the notion that procedural learning, rooted in the dopamine rich basal ganglia, plays an important role in language development [57]–[59].

The relationship between the neighboring genes *ANKK1* and *DRD2* has been a source of controversy. In our study, we found association between language and a haplotype block stretching across *ANKK1* and *DRD2*, suggesting that we, like most studies, are unable to refine our associations to a single gene. However, previous work has shown that the rs1800497 polymorphism is associated with the number of D2 dopamine binding sites and glucose metabolism in the central nervous system [60]–[62]. Reduced dopamine signaling and glucose metabolism may adversely affect high order cognitive functioning including verbal language processing. Functionally, there is limited evidence on how *ANKK1* and *DRD2* may interact. Huang et al. suggested *ANKK1* may influence *DRD2* expression via NF-kB signaling [63]. However, evidence supporting this hypothesis is limited and *in vivo* analyses are needed to discern any functional relationship between *ANKK1* and *DRD2*. Additionally, associations of *ANKK1* and *DRD2* may reflect linkage disequilibrium in the locus, and may be capturing the signal from a single, unidentified causative variant.

### Limitations

This investigation is subject to several limitations. First, the use of maximum amount of prenatal nicotine exposure may be an overestimation due to possible reductions and cessations of smoking during the prenatal period. However, the smoking data obtained accurately reflects the exposure since the information was collected in the pre/perinatal period. Second, although we controlled for many factors associated with language, this study cannot control for all possible, unmeasured factors that may confound associations. However, our models encompass a broad range of covariates relative to other previous studies. Third, due to the design of the ALSPAC cohort and amount of time following subjects, missing data are to be expected. The subsample used to complete association analyses has various demographic and environmental differences compared to the overall sample, which is more representative of the general population in the Avon region of the United Kingdom (Table S2). These factors were controlled for in the analysis of prenatal nicotine exposure, but our findings must be replicated in a more diverse, representative sample before being expanded to the general population. Fourth, there are inherent differences between our discovery cohort, ALSPAC, and our replication cohort, Iowa LI. Subjects in ALSPAC were recruited during the prenatal period, and investigators aimed to collect a sample that reflected the general population in the Avon region of the United Kingdom. Iowa LI is a case-control cohort that recruited cases with LI and matched controls. Therefore, genetic associations of *ANKK1*/*DRD2* in the two cohorts are not identical. However, the initial and replicated associations do suggest that *ANKK1*/*DRD2* and dopamine signaling modulate language skills in children.

## Conclusions

Prenatal nicotine exposure has a negative effect on language abilities in schoolchildren. These results support the growing body of evidence that the development of communication skills begins during fetal development. Future studies should determine the effects of exposure to first-hand nicotine exposure and other prenatal and postnatal toxins. The genetic associations of *ANKK1* and *DRD2* with language performance further suggest that nicotine-related pathways modulate verbal language processing. More specifically, we implicate dopamine signaling in the comprehension and processing of verbal language. Other factors in dopamine and other major neurotransmitter signaling pathways should be examined.

## Supporting Information

Table S1 **Distribution of covariates among smoking groups.** Values are either percentages or means (SD). *Indicates χ^2^ two-tailed p-value <0.05 from univariate analyses of each covariate and prenatal nicotine exposure outcome. **Indicates ANOVA p-value <0.05 from comparison of each covariate and prenatal nicotine exposure outcome.
(DOC)

Table S2 **Comparison of those included in analyses and the overall ALSPAC cohort. Data are presented as either percentages or mean (SD).**
(DOC)
