# Supplementary material for: Associations of Prenatal Nicotine Exposure and the Dopamine Related Genes ANKK1 and DRD2 to Verbal Language
Source: PLoS One. 2013 May 15;8(5):e63762. doi: 10.1371/journal.pone.0063762 (PMC3655151; doi:10.1371/journal.pone.0063762)
Supplement: Table S1 — Distribution of covariates among smoking groups. Values are either percentages or means (SD). *Indicates χ2 two-tailed p-value <0.05 from univariate analyses of each covariate and prenatal nicotine exposure outcome. **Indicates ANOVA p-value <0.05 from comparison of each covariate and prenatal nicotine exposure outcome. (DOC) [file pone.0063762.s001.doc]

**Table S1**

| **Variable** |  | **Smoking Exposure** | | | |  |
| --- | --- | --- | --- | --- | --- | --- |
|  |  | **None** | **Any** | **Low** | **High** | **Overall** |
| **Sex** | Male | 49.4 | 51.8 | 52.6 | 48.6 | 49.7 |
|  | Female | 50.6 | 48.2 | 47.4 | 51.4 | 50.3 |
| **Resuscitation** | Yes | 8.4 | 6.6 | 6.7 | 6.1 | 8.2 |
| **Antenatal class attendance*** | Yes | 67.0 | 57.9 | 60.2 | 48.2 | 65.7 |
| **Gestational age** | <36 wks | 5.3 | 5.5 | 5.5 | 5.6 | 5.3 |
|  | >37 wks | 94.7 | 94.5 | 94.5 | 94.4 | 94.7 |
| **ADHD** | Yes | 1.5 | 2.3 | 2.1 | 3.5 | 1.6 |
| **Maternal social class*** | Non-manual | 13.2 | 29.1 | 26.4 | 43.1 | 15.2 |
|  | Manual | 86.2 | 70.9 | 73.6 | 56.8 | 84.8 |
| **Alcohol Consumption*** | None | 44.1 | 37.6 | 37.6 | 37.8 | 43.2 |
|  | 1 PWK | 41.6 | 39.7 | 39.7 | 40.0 | 41.4 |
|  | 1+ PWK | 13.1 | 19.0 | 18.9 | 19.3 | 13.9 |
|  | 1+ PDAY | 1.2 | 3.6 | 3.8 | 3.0 | 1.5 |
| **Type of school child attends*** | Primary | 91.8 | 95.0 | 94.7 | 96.5 | 92.2 |
|  | Other | 2.4 | 1.6 | 1.3 | 2.6 | 2.3 |
|  | Private | 5.8 | 3.4 | 4.0 | 0.9 | 5.5 |
| **Hours interacting with child** | <30 min | 12.3 | 14.0 | 14.0 | 13.9 | 12.5 |
|  | 30-60 min | 43.2 | 42.5 | 43.9 | 36.5 | 43.2 |
|  | 1-2 hrs | 35.5 | 36.6 | 34.9 | 44.3 | 35.7 |
|  | 3+ hrs | 8.9 | 6.9 | 7.3 | 5.2 | 8.6 |
| **Birth-weight** (g)** |  | 3461.94 (526.4) | 3296.55 (521.0) | 3312.04 (518.4) | 3229.02 (528.8) | 3439.11 (528.7) |
| **Mother’s Age** (yr)** |  | 29.67 (4.3) | 27.6 8 (4.8) | 27.96 (4.9) | 27.7 5 (4.5) | 29.3 (4.4) |
|  |  |  |  |  |  |  |
