# Supplementary material for: Associations of Prenatal Nicotine Exposure and the Dopamine Related Genes ANKK1 and DRD2 to Verbal Language
Source: PLoS One. 2013 May 15;8(5):e63762. doi: 10.1371/journal.pone.0063762 (PMC3655151; doi:10.1371/journal.pone.0063762)
Supplement: Table S2 — Comparison of those included in analyses and the overall ALSPAC cohort. Data are presented as either percentages or mean (SD). (DOC) [file pone.0063762.s002.doc]

**Table S2**

| **Variable** |  | **Sample 1**  **N=5579** | **Overall**  **N=15221** |
| --- | --- | --- | --- |
|  |  |
| **Sex** | Male | 49.7 | 51.4 |
|  | Female | 50.3 | 48.6 |
| **Resuscitation** | Yes | 8.2 | 9.2 |
| **Antenatal Class Attendance** | Yes | 65.6 | 60.0 |
| **Gestational Age** | <36 wks | 5.3 | 9.6 |
|  | >37 wks | 94.7 | 90.4 |
| **ADHD** | Yes | 1.6 | 2.1 |
| **Social Class-Maternal** | Manual | 15.4 | 19.8 |
|  | Non-manual | 84.6 | 80.2 |
| **Maternal Alcohol Consumption** | None | 43.3 | 45.5 |
|  | 1 PWK | 41.3 | 38.7 |
|  | 1+ PWK | 13.9 | 13.9 |
|  | 1+ Pday | 1.5 | 2.0 |
| **Ethnicity** | White | 100.0 | 97.4 |
|  | Non-white | 0.0 | 2.6 |
| **Type of school child attends** | Primary | 92.2 | 91.9 |
|  | Other | 2.3 | 2.9 |
|  | Private | 5.5 | 5.3 |
| **Hours interacting with child** | None | 0.0 | 0.2 |
|  | <30 min | 12.5 | 13.3 |
|  | 30-60 min | 43.1 | 42.6 |
|  | 1-2 hrs | 35.7 | 35.2 |
|  | 3+ hrs | 8.6 | 8.7 |
| **Birth-weight (g)** |  | 3437.5 (530.5) | 3381.5 (580.8) |
| **Mother’s Age at Delivery (yr)** |  | 29.3 (4.4) | 28.0 (5.0) |
